# Uterine ERBB3 signaling is critical to cultivate stromal environment for functional gland branching

**DOI:** 10.1101/2025.10.16.682855

**Published:** 2026-10-30

**Authors:** Bo Li, Amanda Dewar, Sudhansu K Dey, Xiaofei Sun

## Abstract

Preimplantation uterine gland differentiation, involving glandular branching, is a prerequisite for secretion of leukemia inhibitory factor (LIF), thereby establishing uterine receptivity to embryo implantation. This differentiation process is disrupted by the absence of FOXA2, a transcription factor specifically present in uterine glands. It remains unclear whether preimplantation glandular differentiation is regulated exclusively by gland-intrinsic mechanisms or is influenced by the surrounding microenvironment through mesenchymal-epithelial interactions. We show here that uterine deletion of Erbb3 leads to impaired gland branching and FOXA2 expression, as well as reduced female fertility. In contrast, females with uterine epithelial-specific deletion of Erbb3 show normal fertility, suggesting a critical role of stromal ERBB3 signaling. A transcriptomic profiling of day 3 stromal cells identified Igf1 as a candidate paracrine factor mediating the stromal-epithelial interaction. Thus, the uterine deletion of Igf1r leads to a reduced gland branching phenotype similar to that observed in Erbb3d/d uteri. These results demonstrate that ERBB3 regulates glandular branching and embryo implantation via the ERBB3-IGF1-IGF1R signaling pathway.

## Full Text Availability

The license terms selected by the author(s) for this preprint version do not permit archiving in PMC. The full text is available from the preprint server.

